# Administration of alpha-ketoglutarate improves epithelial restitution under stress injury in early-weaning piglets

**DOI:** 10.18632/oncotarget.20555

**Published:** 2017-08-24

**Authors:** Liuqin He, Xihong Zhou, Niu Huang, Huan Li, Zhijie Cui, Junquan Tian, Qian Jiang, Shaojuan Liu, Jian Wu, Tiejun Li, Kang Yao, Yulong Yin

**Affiliations:** ^1^ Key Laboratory of Agro-ecological Processes in Subtropical Region, Institute of Subtropical Agriculture, Chinese Academy of Sciences, Scientific Observing and Experimental Station of Animal Nutrition and Feed Science in South-Central, Ministry of Agriculture, Hunan Provincial Engineering Research Center for Healthy Livestock and Poultry Production, Changsha, Hunan 410125, China; ^2^ University of Chinese Academy of Sciences, Beijing, 10039, China; ^3^ College of Animal Science and Technology, Hunan Agricultural University, Changsha, Hunan, 410128, China; ^4^ Xiangtan University, Xiangtan, Hunan 411105, China; ^5^ Hunan Co-Innovation Center of Animal Production Safety, Changsha, Hunan, 410128, China; ^6^ Laboratory of Animal Nutrition and Human Health, College of Life Sciences, Hunan Normal University, Changsha, Hunan, 410006, China

**Keywords:** AKG, inflammatory cytokines, nutrient-sensing transporters, tight-junction proteins, epithelial restitution

## Abstract

Alpha-ketoglutarate (AKG) is an important cellular metabolite that participates in energy production and amino acid metabolism. However, the protective effects and mechanism of AKG on mucosal lesions have not been well understood. This study was conducted to investigate the effects of dietary AKG supplementation on epithelial restitution in early-weaning piglets under *Escherichia coli* lipopolysaccharide (LPS) induction. A total of 32 weaned piglets were used in a 2 × 2 factorial design; the major factors were dietary treatment (basal diet or AKG diet) and inflammatory challenge (LPS or saline). The results showed that AKG supplementation improved the growth performance and intestinal morphology in the LPS-induced early-weaning piglets. Compared with the basal diet, the AKG diet remarkably decreased the concentration and mRNA expression of intestinal inflammatory cytokines (IL-1β, IL-6, and IL-12) in the LPS-induced piglets. Moreover, AKG administration upregulated the mRNA expression of nutrient-sensing transporters (GLUT-2, SGLT-1, PEPT-1, I-FABP2) in the small intestine of both saline- and LPS-treated piglets, and improved the distribution and expression of tight-junction genes andproteins (ZO-1, Occludin, Claudins, E-cadherin). Collectively, our findings indicate that AKG has the potential to alleviate intestinal inflammatory response and improve epithelial restitution and nutrient-sensing ability under stress injury in early-weaning piglets, and it also provides an experimental basis for enteral use of AKG in swine production and clinical application to prevent intestinal epithelial damage.

## INTRODUCTION

Intestinal epithelial barrier restitution plays a key role in the maintenance of gastrointestinal tract (GIT) homeostasis under stress conditions [1]. Stresses such as bacteria, viruses, environmental factors and diets, may lead to increased epithelial permeability and then cause intestinal dysfunction and impair immunity response in mammals [2]. Eventually, these stresses would trigger a multitude of gastrointestinal disorder diseases including inflammatory bowel diseases (IBD), irritable bowel syndrome (IBS), and infectious diarrhea [3]. There is clear evidence that adequate nutrition is a key factor in the maintenance of epithelial restitution and barrier protection against potential risk [4]. Thus, nutritional support (*e.g*., amino acids) can help maintain integrity of barrier structure in animals and human with intestinal diseases [5]. Emerging evidence has shown that alpha-ketoglutarate (AKG), an endogenous intermediary metabolite in the tricarboxylic acid (TCA) cycle, is a signaling molecule involved in multiple metabolic and cellular pathways [6] and may be beneficial for gut integrity [7, 8]. Recent reports have also suggested that AKG may improve intestinal immunity and inhibit apoptosis in response to inflammatory stimuli [9, 10].

Tight junctions (TJs) are known to be an important indicator of intestinal health, and are considered to one of the properties of epithelial restitution [11]. Alterations in TJs structure and function have been found in intestinal stress injury [12]. A recent study showed that a reduction of the key TJ proteins, such as zonula occludens (ZOs), Occludin, and Claudins, leads to an increase in intestinal permeability and a decrease in nutrient transport [13]. To date, few reports document that AKG at the site of mucosal lesions could attenuate intestinal stress injury through improving epithelial restitution and nutrient transport ability. Therefore, we conducted the present study to investigate whether AKG as a potent feed additive agent could improve the expression of TJs and nutrient-sensing transporters under stress injury in early-weaning piglet.

## RESULTS

### Growth performance

During the pre-challenge period (1~21d), AKG supplementation did not affect the final body weight (BW), average daily gain (ADG) and the ratio of gain to feed (G:F) compared with piglets fed the basal diets (Table 1). However, in the first weaning week (1~7d), piglets fed the AKG diet had greater average daily feed intake (ADFI) and ADG than those fed the basal diet. To simulate stress injury, we intraperitoneally administrated piglets with *Escherichia coli* lipopolysaccharide (LPS). During the LPS challenge period (22~ 30d), LPS challenge significantly decreased ADG and ADFI in piglets fed both the basal diet and AKG diet, while AKG supplementation increased ADG and ADFI in the LPS-induced piglets. A LPS challenge × diet interaction was observed for ADFI and G:F. In the overall experiment period, compared with the LPS-induced piglets fed the basal diet, the values of final BW, ADG, and ADFI in pigs fed the AKG diet were higher.

**Table 1:** Effects of AKG administration on the growth performance of early-weaning piglets

Item	Basal diet		AKG diet		Pooled	P-value		
	CON	CON+LPS	AKG	AKG+LPS	SEM	Diet	LPS	Diet×LPS
Body weight, kg								
Day 0	6.20	6.20	6.19	6.21	0.11	0.949	0.934	0.935
day 7	6.80	6.82	7.38	7.30	0.12	0.055	0.689	0.965
day 14	8.12	8.21	8.65	8.87	0.14	0.114	0.956	0.471
day 21	11.08	11.11	11.3	11.70	0.16	0.219	0.507	0.561
day 30	15.27	13.43	15.47	14.10	0.26	0.325	0.001	0.587
Average daily gain, g/d								
day 0 to 7	90.48	102.38	137.62	138.33	6.28	0.001	0.544	0.590
day 7 to 14	213.42	218.37	251.65	250.49	4.73	0.001	0.572	0.734
day 14 to 21	385.51	408.57	405.47	402.45	6.72	0.621	0.476	0.355
day 0 to 21	232.70	240.82	262.72	266.67	7.24	0.062	0.676	0.885
day 22 to 30	442.26	234.98	469.44	345.20	19.95	0.381	<0.0001	0.616
day 0 to 30	301.91	226.6	306.67	273.89	8.79	0.064	0.001	0.126
Average daily feed intake, g/d								
day 0 to 7	199.20	207.50	216.04	228.98	4.16	0.002	0.534	0.750
day 7 to 14	319.64	321.43	347.62	354.76	7.49	0.127	0.536	0.587
day 14 to 21	545.35	566.36	545.23	575.24	8.62	0.391	0.052	0.741
day 0 to 21	343.07	365.4	382.94	413.12	9.09	0.012	0.116	0.809
day 22 to 30	732.65	558.17	745.00	603.11	21.88	0.001	<0.0001	0.032
day 0 to 30	467.7	410.49	504.74	443.4	9.55	0.034	0.0003	0.996
Gain: Feed, g/g								
day 0 to 7	0.48	0.47	0.54	0.57	0.08	0.052	0.893	0.539
day 7 to 14	0.70	0.66	0.64	0.62	0.04	0.318	0.409	0.820
day 14 to 21	0.68	0.72	0.70	0.68	0.03	0.673	0.646	0.296
day 0 to 21	0.67	0.65	0.61	0.65	0.03	0.329	0.809	0.411
day 22 to 30	0.60	0.40	0.64	0.57	0.09	0.004	0.001	0.022
day 0 to 30	0.62	0.54	0.61	0.58	0.05	0.704	0.121	0.539

Values are LSmean plus pooled SEM, n=8.

### Small intestinal length and morphology

**Figure 1:**
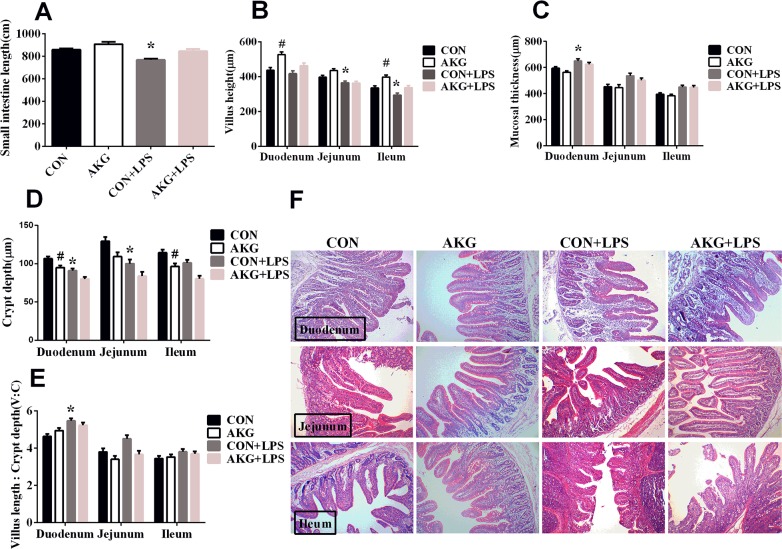
Effects of AKG administration on intestinal morphology of early-weaning piglets Values are LSmean plus pooled SEM, n=8. ^*^Indicates a statistically significant difference for challenge (saline or LPS) (P < 0.05). ^#^ Indicates a statistically significant difference for dietary treatment (basal or AKG) (P < 0.05). CON, control piglets; AKG, piglets treated with AKG; CON + LPS, piglets treated with LPS; AKG and LPS, piglets treated with LPS and AKG. **(A)** Small intestine length. **(B)** Villus height. **(C)** Mucosal thickness. **(D)** Crypt depth. **(E)** The ratio of villus length and crypt depth. **(F)** Representative image (×100) of intestinal morphology.

Intestinal morphology is the major indicator of intestinal health and also reflects the maturation rate of enterocytes [14]. Small intestine (Figure 1A) in the piglets fed the AKG diet was longer than those fed the basal diet. AKG supplementation exhibited an increased villus height (Figure 1B) in the duodenum, jejunum, and ileum, and a decreased mucosal thickness (Figure 1C), crypt depth (Figure 1D), and villus length: crypt depth (V: C) (Figure 1E) in the LPS-induced piglets. These results suggest that AKG could improve intestinal morphology (Figure 1F) under stress injury in the early-weaning piglets.

### The secretion and mRNA expression of inflammatory cytokines in intestine

**Figure 2:**
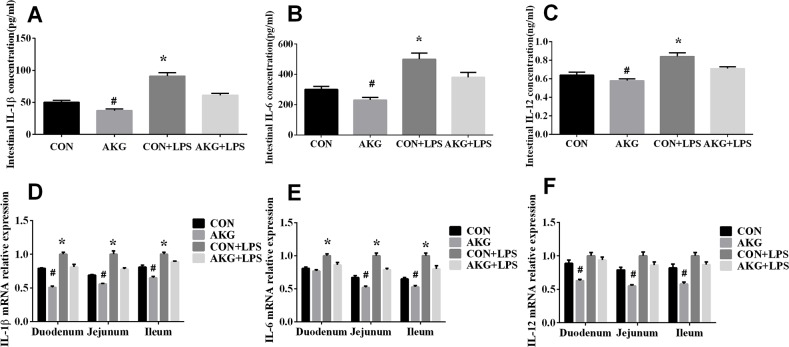
Effects of AKG administration on the concentration and mRNA expression of intestinal inflammatory cytokines in the early-weaning piglets **(A)** Intestinal IL-1β concentration. **(B)** Intestinal IL-6 concentration. **(C)** Intestinal IL-12 concentration. **(D)** IL-1β mRNA abundance in small intestine. **(E)** IL-6 mRNA abundance in small intestine. **(F)** IL-12 mRNA abundance in small intestine.

When early-weaning piglets were treated with LPS, the concentrations and mRNA expression of interleukin 1β (IL-1β), interleukin 6 (IL-6), and interleukin 12 (IL-12) in the small intestine were detected. We found that compared with the LPS-induced piglets fed the basal diet, piglets fed the AKG diet significantly decreased the concentrations of IL-1β (Figure 2A), IL-6 (Figure 2B), and IL-12 (Figure 2C) in the intestine. In addition, according to the analysis of the real-time qPCR, AKG supplementation also reduced the mRNA expression of IL-1β (Figure 2D), IL-6 (Figure 2E), and IL-12 (Figure 2F) in the duodenum, jejunum, and ileum in the LPS-induced piglets, suggesting the improvement of intestinal inflammation by AKG administration.

### mRNA expression of nutrient-sensing transporters in small intestine

**Figure 3:**
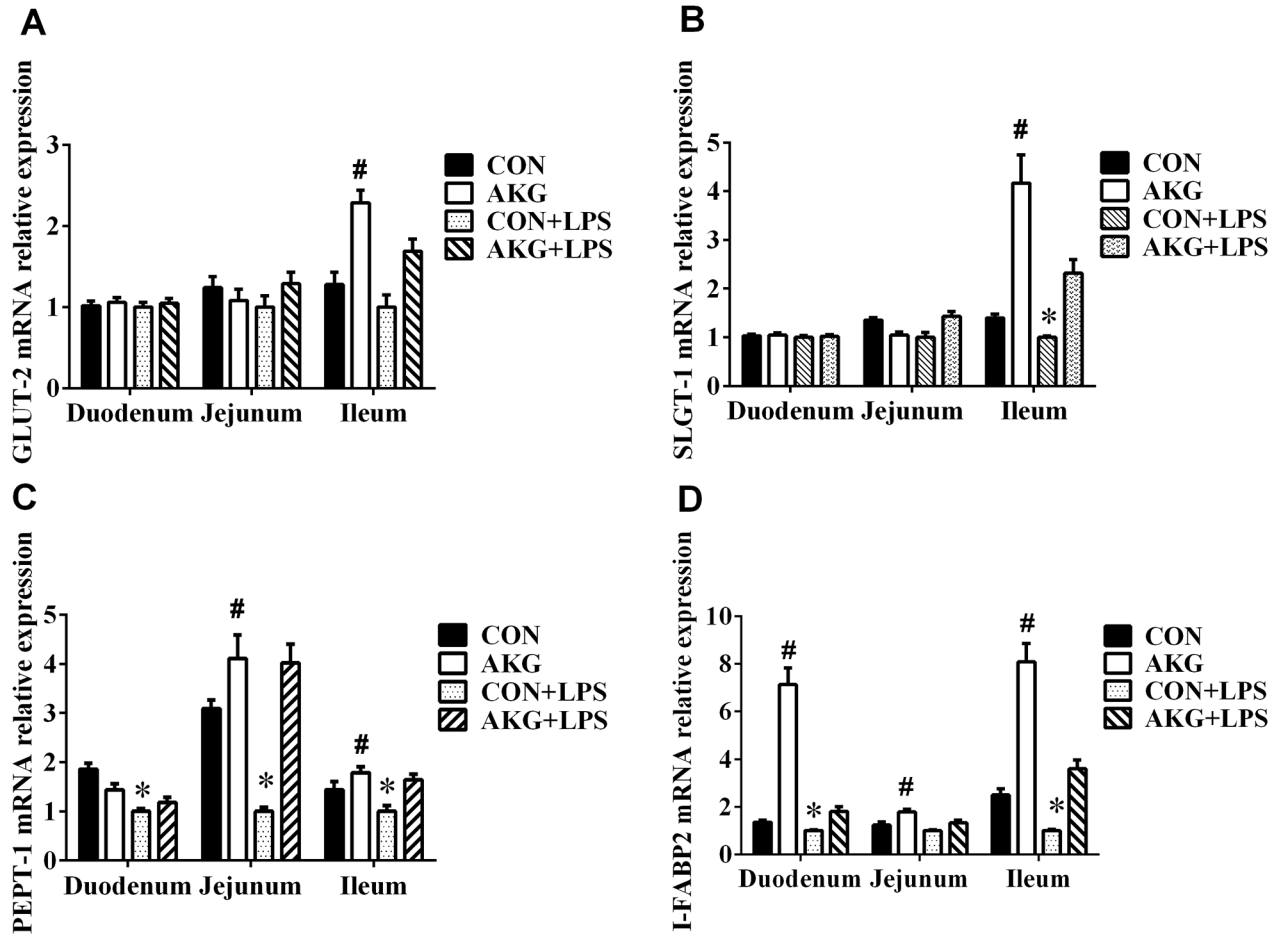
Effects of AKG administration on the mRNA expression of nutrient-sensing transporters in the small intestine of piglets **(A)** GLUT-2 mRNA abundance. **(B)** SGLT-1 mRNA abundance. **(C)** PEPT-1 mRNA abundance. **(D)** I-FABP2 mRNA abundance.

Nutrient chemosensing system involved in nutrient-sensing transporters plays a vital role in the regulation of various nutrient (*e.g*., glucose, peptide, fatty acids) transport and absorption. To investigate whether AKG rectify impaired intestinal nutrient transport, we determined the mRNA expression of glucose, peptide and fatty acid transporters in the small intestine of early-weaning piglets under stress injury. We found that AKG supplementation increased the mRNA expression of facilitated glucose transporter 2 (GLUT-2) (Figure 3A) and Na^+^ coupled glucose transporter 1 (SGLT-1) (Figure 3B) in the ileum of piglets treated with either LPS or saline. A remarkable reduction was observed in the mRNA expression of peptide transporter-1(PEPT-1) (Figure 3C) and intestinal fatty acid binding protein 2 (I-FABP2) (Figure 3D) in the LPS-induced piglets. However, AKG administration enhanced the mRNA expression of PEPT-1 in the jejunum and ileum of pigs, as well as I-FABP2 mRNA abundance in the small intestine. These suggest that AKG administration could help promote the absorption and transport of nutrients in the intestine of early-weaning piglets under stress injury.

### Location and expression of tight junctions in small intestine

Alterations in tight-junction structure and function are known to be an important indicator of epithelial restitution [13]. Thus, to validate whether AKG could promote intestinal epithelial restitution under stress injury, we determined the expression and location of TJs in the small intestine using the real-time qPCR, Immunohistochemical, and Western blot analysis. We found that in the duodenum, LPS challenge decreased the mRNA abundance of zonula occluden-1 (ZO-1), Occludin, and Claudin-1 in piglets fed both the basal diet and AKG diet, but had no effect on the mRNA expression of E-cadherin, Claudin-2, Claudin-3, Claudin-4, Claudin-5, Claudin-6, and Claudin-7 (Table 2). In the jejunum, AKG supplementation increased the mRNA abundance of Occludin, Claudin-1, Claudin-6, ZO-1, and E-cadherin in the LPS-induced piglets. In the ileum, the mRNA abundance of Occludin, E-cadherin, Claudin-1, Claudin-2, Claudin-3, Claudin-4, Claudin-6, and Claudin-7 in piglets fed the AKG diet was increased in response to LPS challenge. There was an LPS challenge × diet interaction (P < 0.05) on the mRNA abundance of Claudin-1, Claudin-3 and Claudin-7 in the jejunum and ileum.

**Table 2:** Effects of AKG administration on the mRNA expression of tight-junction genes in the small intestine of early-weaning piglets

Item	Basal Diet		AKG Diet		Pooled	P-value		
	CON	CON+LPS	AKG	AKG+LPS	SEM	Diet	LPS	Diet×LPS
Duodenum								
ZO-1	1.56	1.00	2.33	0.85	0.14	0.100	<0.0001	0.020
Occludin	1.55	1.00	3.89	1.39	0.27	0.0001	<0.0001	0.003
E-cadherin	1.09	1.00	1.10	0.99	0.05	0.971	0.403	0.934
Claudin-1	2.54	1.00	7.56	2.03	0.68	0.001	0.0003	0.02
Claudin-2	1.12	1.00	1.07	1.00	0.05	0.845	0.378	0.841
Claudin-3	1.27	1.00	1.03	1.11	0.06	0.607	0.437	0.194
Claudin-4	1.22	1.00	1.10	1.16	0.06	0.856	0.534	0.275
Claudin-5	0.99	1.00	1.03	1.05	0.07	0.746	0.905	0.957
Claudin-6	1.37	1.00	1.16	0.94	0.09	0.490	0.125	0.715
Claudin-7	1.25	1.00	1.26	1.16	0.07	0.563	0.256	0.645
Jejunum								
ZO-1	1.59	1.00	1.44	1.52	0.09	0.300	0.157	0.069
Occludin	1.71	1.00	2.86	1.88	0.19	0.005	0.016	0.671
E-cadherin	1.19	1.00	1.22	1.17	0.07	0.482	0.387	0.589
Claudin-1	1.46	1.00	4.39	1.76	0.30	<0.0001	<0.0001	0.001
Claudin-2	1.97	1.00	2.12	1.78	0.14	0.067	0.014	0.215
Claudin-3	1.62	1.00	1.02	1.18	0.08	0.136	0.112	0.010
Claudin-4	2.09	1.00	2.12	1.89	0.15	0.099	0.020	0.117
Claudin-5	1.89	1.00	2.08	1.63	0.14	0.093	0.010	0.369
Claudin-6	1.36	1.00	2.05	1.62	0.14	0.015	0.129	0.899
Claudin-7	1.97	1.00	1.68	1.55	0.12	0.524	0.011	0.042
Ileum								
ZO-1	0.79	1.00	0.74	0.78	0.05	0.137	0.160	0.347
Occludin	1.47	1.00	2.66	1.79	0.17	0.001	0.019	0.456
E-cadherin	1.75	1.00	2.01	2.25	0.16	0.012	0.365	0.096
Claudin-1	1.40	1.00	3.42	1.82	0.24	<0.0001	0.002	0.039
Claudin-2	2.14	1.00	4.99	2.93	0.38	<0.0001	0.002	0.307
Claudin-3	1.70	1.00	6.19	1.64	0.54	0.001	0.001	0.008
Claudin-4	1.20	1.00	2.57	1.49	0.20	0.012	0.071	0.206
Claudin-5	1.07	1.00	1.13	1.00	0.06	0.817	0.413	0.791
Claudin-6	2.23	1.00	2.65	2.46	0.21	0.009	0.041	0.130
Claudin-7	1.78	1.00	1.84	2.30	0.15	0.011	0.515	0.019

Values are LSmean plus pooled SEM, n=8.

**Figure 4:**
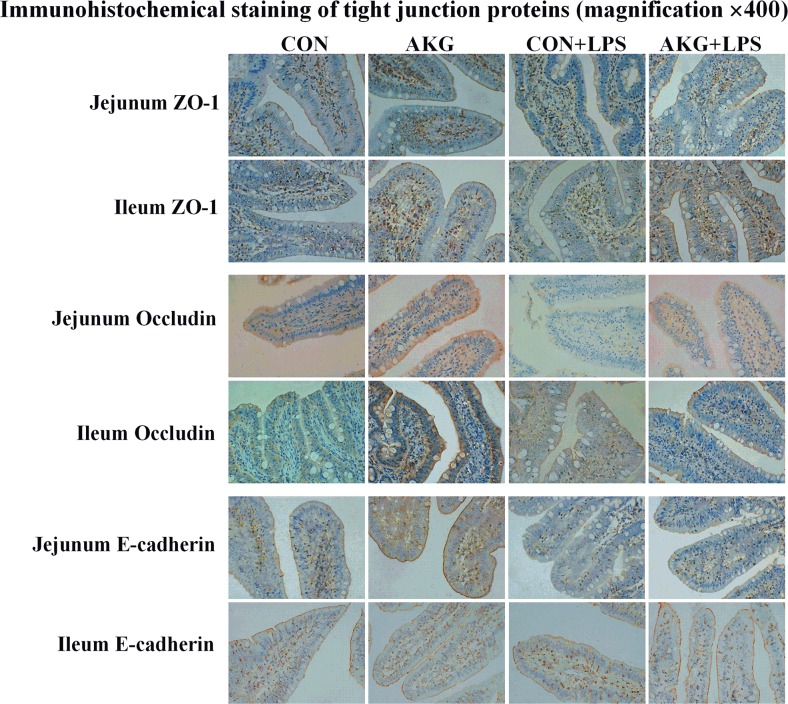
Effects of AKG administration on representative images of immunohistochemical staining (magnification × 400) of the tight-junction proteins in the jejunum and ileum of early-weaning piglets using the Immunohistochemical analysis

To further investigate whether AKG could stimulate epithelial restitution *in vivo*, the location and expression of TJ proteins were determined. Results of the immunohistochemical analysis indicated that ZO-1, Occludin, and E-cadherin proteins were mostly located in the epithelial cell membrane of jejunum and ileum, partly in cytoplasm (Figure 4). Moreover, LPS challenge disrupted the distribution of ZO-1, Occludin, and E-cadherin proteins in the jejunum and ileum, however, AKG administration protected the original function of TJs against stress injury and improved the rebuilding of impaired TJs.

**Figure 5:**
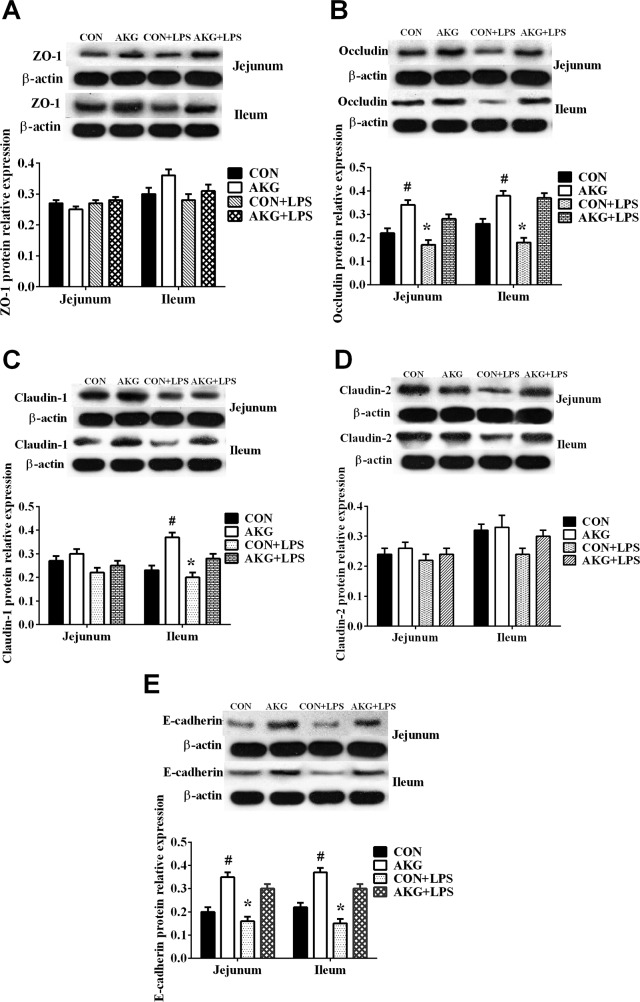
Effects of AKG administration on the expression of tight-junction proteins in jejunum and ileum of early-weaning piglets using the Western blot technique **(A)** The relative expression of ZO-1 protein. **(B)** The relative expression of Occludin protein. **(C)** The relative expression of Claudin-1 protein. **(D)** The relative expression of Claudin-2 protein. **(E)** The relative expression of E-cadherin protein.

In addition, results of the western blot analysis showed that LPS challenge remarkably decreased the protein expression of Occludin (Figure 5B) and E-cadherin (Figure 5E) in the jejunum and ileum of piglets fed both the basal diet and AKG diet, and also decreased Claduin-1 (Figure 5C) expression in the ileum. However, AKG supplementation increased the protein expression of Occludin and E-cadherin in the jejunum and ileum of both saline- and LPS-treated piglets as well as the protein expression of Claduin-1 in the ileum. Neither AKG supplementation nor LPS challenge affected the expression of ZO-1 (Figure 5A) and Claudin-2 (Figure 5D) proteins in the jejunum and ileum.

## DISCUSSION

Alpha-ketoglutarate is considered to be a potent therapeutic agent against GIT disorder diseases [15] and also has potential to implicate as a new feed additive for weaning piglets [16]. Many reports have suggested that maternal and neonatal administration of AKG could improve the growth performance of piglets [7, 17, 18]. In the present study, dietary supplementation with 1% AKG remarkably increased BW, ADG and ADFI of early-weaning piglets during the first feeding week (1~7d), but the final BW, ADG and G:F had no significant change after AKG administration (1~21d). These results indicate that AKG administration might alleviate the effects of weanling stress on growth performance while it had no growth-promoting effects in young growing piglets under normal conditions. After LPS challenge (22~30d), ADG and ADFI in piglets fed the AKG diet were greater than those fed the basal diet, suggesting that AKG might protect piglets from LPS-induced stress. Pervious study demonstrated that the beneficial effects of AKG on the growth performance of piglets under LPS stimuli may be associated with its capacity of improving gut immunity and protein synthesis [19]. In addition, AKG, as an endogenous intermediary metabolic in the TCA cycle and a precursor in the amino acid biosynthesis, plays an important role in lipids, amino acids, and nucleic acids metabolism [20], and help resist stress injury by regulating ATP production [21].

The small intestine is the main organ for nutrient digestion and absorption in mammals. Villus height and crypt depth reflect the number and maturation rate of enterocytes, which affect the absorption and transport ability of nutrients in intestine [22]. The present results showed an increase in villus height and a decrease in crypt depth and mucosal thickness in the small intestine of piglets fed the AKG diet. These results were consistent with the pervious study [7], suggesting that AKG in enterocytes may beneficially regulate the intracellular endogenous amino acid concentrations via TCA cycle [23] and then influence various signaling pathways [16], such as the AMP-activated protein kinase (AMPK), nuclear factor kappa B (NF-κB), and mammalian target of rapamycin (mTOR) pathways, thereby improving intestinal morphology.

One of our recent studies demonstrates that AKG exert beneficial effects on the modulation of inflammatory cytokines such as IL-2 IL-8, IL-10, TGF-β, TNF-α and IL-17 [24], which have a negative influence on gut integrity and epithelial restitution when excessively secreted [6]. This is consistent with our findings that the addition of AKG inhibited the secretion and mRNA expression of IL-1β, IL-6, and IL-12 in the small intestine of LPS-induced piglets, indicating the possible anti-inflammatory effect of enteral supply of AKG during stress injury. Previously, Garrett-Cox and co-workers found that glutamine suppressed plasma TNF-α and IL-10 concentrations in the LPS-induced rat [25], while some other researchers observed that glutamine blunted the increased secretion of TNF-α in the intestine but not in the plasma [26]. Recently, Chen et al (2015) reported that AKG lowered body weight and affected intestinal innate immunity through influencing intestinal microbiota in mice [27]. Of note, our group also found that AKG could suppress NF-κB-mediated inflammatory pathway and enhance the PXR-regulated detoxification pathway to improve intestinal immune and self-detoxification system [24, 28]. It is further confirmed by our study that AKG as a precursor for the biosynthesis of glutamate and glutamine, inproved the secretion and expression of inflammatory cytokines in response to LPS stimulation, thereby increasing intestinal immunity.

The transport and absorption of nutrients including glucose, fatty acids, and peptides, is influenced by gut nutrient chemosensing system including various transporters and channel proteins in mammals [14]. It is evident that glucose, peptide, and fatty acid transporters play a vital role in controlling the utilization of nutrients during digestion [29]. Previous studies reported that the absorption of glucose mainly relies on two types of transporters in intestine [30], Na^+^ coupled glucose transporters (*e.g*., SGLT-1) on the apical membrane and facilitated glucose transporters (*e.g*., GLUT-2) that regulate the basolateral exit of glucose. Glucose uptake depends on the presence of SGLT-1, which can transport both glucose and galactose [31]. GLUT-2 could mediate the balance of the glucose concentration under a lower glucose condition [32], but it is dispensable for normal intestinal glucose absorption when SGLT-1 is activated. In our current study, under stress injury conditions, an improvement in the mRNA abundance of GLUT-2 and SGLT-1 was observed by AKG administration in the ileum of early-weaning piglets, suggesting the regulation of AKG in the glucose absorption and transport. Interestingly, the addition of AKG also enhanced the expression mRNA of PEPT-1 and I-FABP2 in the LPS-induced piglets. Small peptides (i.e., di- and tri-peptide) are absorbed into intestinal epithelial cells via a variety of membrane transport systems (*e.g*., PEPT-1) or degraded to various amino acids [33]. Free fatty acids are not only an important directed source of energy in pigs but also play key roles in regulating a series of physiological responses [34]. Thus, these results suggest that AKG supplementation might improve the absorption and transport of nutrients via promoting the expression of nutrient-sensing transporters in the piglets under stress injury.

Several lines of evidence that support a link between tight-junction proteins and epithelial restitution [35]. TJs are the most apical intercellular structures in epithelial and endothelial cells, including transmembrane proteins (*e.g*., Claudins, Occludin, E-cadherin) and cytosolic proteins (*e.g*., ZO-1) [36], which regulate lateral intercellular permeability and are crucial for the integrity of the epithelial barrier [37]. Numerous LPS-induced animal studies have shown that the expression of TJ proteins may also be an important factor in evaluating the extent of tissues damage [3]. In our present study, we found that ZO-1, Occludin, and E-cadherin proteins were mostly located in the intestinal epithelial cell membrane of jejunum and ileum of early-weaning piglets by using the immunohistochemical analysis. LPS challenge disrupted their original distribution and then resulted in the dysfunction of intestinal barrier. While AKG supplementation improved the rebuilding of these TJ proteins. Furthermore, AKG could upregulate the mRNA and protein expression of TJs in the intestine of early-weaning piglets. Many previous studies reported that a decrease in tight junction expression could lead to alteration or disruption of intestinal barrier [38]. The reduction of Occludin, Claudins, ZOs and other tight junctions may induce IBD, IBS and infectious diarrhea [39]. The decreased AKG synthesis has recently been demonstrated in IBD patients with increased intestinal permeability associated with the altered expression of TJs [40]. It is evident that LPS has been widely used to imitate a stressful event and impairs the intestinal epithelial barrier by downregulating or redistributing TJs [41]. Our results showed that LPS challenge remarkably decreased the expression of Occludin, Claudin-1, and E-cadherin proteins as pervious studies described [42]. Some mechanisms widely proposed that the crosstalk in TJs could promote epithelial restitution or restrict the uncontrolled entry of luminal antigens to maintain intestinal health [43]. As expected, AKG administration increased the expression of Occludin, Claudin-1, and E-cadherin proteins in the intestine of LPS-induced piglets, suggesting that AKG administration strengthens the protective and restorative function of some TJ proteins to defend stress injury. Recent studies with mammals have indicated that AKG used as a dietary supplement can confer favorable effects on both injured and healthy organisms [15, 44]. Mechanically, AKG and its metabolites (i.e., glutamine, glutamate) via the TCA cycle could also help restore intestinal permeability via regulating the expression of water channels and ion transporters and activate AMPK pathway to generate a large number of ATP for cellular osmotic homeostasis under diarrhea conditions [16]. Akobeng et al.(2016) demonstrated that glutamine might lead to the increase of intestinal-mucosal mass and the improvement of TJs under stressful conditions [45]. Additionally, it has been detected that AKG administration positively modulates antioxidant ability and protein synthesis to improve epithelial restitution in rats under protein deficiency and oxidative stress [15, 46].

In summary, our results suggested that AKG-supplemented diet improved growth performance in the first weaning week and maintained intestinal morphology and TJs function in the early-weaning piglets under LPS challenge. Moreover, AKG administration also alleviated inflammatory response and improved nutrient transport to cope with stress injury. These observations provide an experimental basis for enteral use of AKG in swine production and clinical application to prevent intestinal epithelial damage.

## MATERIALS AND METHODS

### Animals and experimental design

This study was approved by the animal welfare committee of the Institute of Subtropical Agriculture, Chinese Academy of Sciences (2013020; Changsha, China). Thirty-two cross-bred (Duroc ×Landrace ×Yorkshire; average body weight (BW) = 6.20 ± 0.11 kg) healthy piglets weaned at 28 days were randomly assigned to 4 treatments (8 pigs/treatment). The experiment was arranged as a 2×2 factorial design. The main factors were dietary treatment (piglets were fed the basal diet or the 1% AKG-supplemented diet) and LPS challenge (piglets were induced with *Escherichia coli* lipopolysaccharide (*E. coli* LPS) or treated with sterile saline by intraperitoneal administration). The composition and nutrient levels of the diets met the nutrient specifications for 5 to 10 kg BW pig according to recommendations of the NRC (2012) (Table 3). After 3 days of adaptation, piglets were fed the experimental diets 3 times per day at 8:00, 12:00 and 17:00. Notably, at 10:00 am on days 22, 25, 28 and 30, piglets in the CON+LPS and AKG+LPS groups were intraperitoneally injected with *E. coli* LPS (100μg/kg BW), respectively, whereas pigs in the CON and AKG groups were injected intraperitoneally with the same volume of sterile saline. During the whole experiment, piglets were housed individually and given free access to water. The final ADG, ADFI and G: F was calculated. The duration of the overall experiment was 30 days. LPS (*Escherichia coli* serotype 055:B5; Sigma Chemical, Inc., St Louis, MO, USA) was dissolved in a sterile physiological solution. The administration dosages of AKG (Wuhan Yuancheng Gongchuang Technology co., LTD, Wuhan, Hubei, China; purity ≥ 99.2%) and LPS were adopted according to the previous experiment [24].

**Table 3:** Ingredients and nutrient composition of the diets

Item	Basal diet (%)	AKG diet (%)
Feed ingredient		
Expanded corn	27.00	27.00
Extruded soybean	15.50	15.50
Maize starch	25.00	24.00
Alpha-ketoglutarate	-	1.00
Dried whey	10.00	10.0.
Plasma protein powder	4.00	4.00
Emulsified oil powder	2.50	2.50
Fish meal	5.00	5.00
98% L-lysine	0.70	0.70
Met	0.20	0.20
L-threonine	0.30	0.30
Trp	0.05	0.05
White sugar	4.00	4.00
Glucose	2.50	2.50
Limestone	0.50	0.50
Dicalcium	0.70	0.70
1% Premix^a^	1.00	1.00
Acidifier	0.90	0.90
Antioxidant	0.05	0.05
Fungicide	0.10	0.1
Total	100	100
Nutrient composition		
Digestible energy MJ/kg	14.02	14.05
Crude protein	20.00	20.00
Lys	1.55	1.55
Met	0.65	0.65
Thr	0.95	0.95
Trp	0.25	0.25
Ca	0.75	0.75
P	0.30	0.30

^a^ Supplied per kilogram of finished feed: vitamin A, 10,800 IU; vitamin D3, 4,000 IU; vitamin E, 40 IU; vitamin K3, 4 mg; vitamin B1, 6 mg; vitamin B2, 12 mg; vitamin B6, 6 mg; vitamin B12, 0.05 mg; biotin, 0.2 mg; folic acid, 2 mg; niacin, 50 mg; D-Calcium pantothenate, 25 mg; Fe, 100 mg as ferrous sulfate; Cu, 150 mg as copper sulphate; Mn, 40 mg as manganese oxide; Zn, 100 mg as zinc oxide; I, 0.5 mg as potassium iodide; and Se, 0.3 mg as sodium selenite.

### Sample collection

On day 30, all pigs were anaesthetized with an intravenous injection of sodium pentobarbital (50 mg/kg BW) and bled by exsanguination. The small intestine was dissected free of the mesentery and sampled on a chilled stainless-steel tray. Ten-cm segments were collected from the middle duodenum, proximal jejunum and distal ileum, respectively, thoroughly flushed with ice-cold phosphate-buffered saline, and then frozen in liquid nitrogen and stored at -80°C for the analysis of gene and protein expression, and one segment was fixed in 10% neutral buffered formalin for examination of intestinal morphology and immunohistochemical analysis.

### Intestinal histomorphology

Paraffin sections (approximately 5 mm) of duodenum, jejunum and ileum samples were stained with hematoxylin and eosin, villus height and crypt depth were measured using a light microscope with a computer-assisted morphometric system (BioScan Optimetric, BioScan Inc., Edmonds, WA, USA). Villus height, mucosal thickness, and crypt depth were defined as in the previous study did [14].

### Determination of intestinal inflammatory cytokines

The physiological concentration of IL-1β, IL-6 and IL-12 in the intestine was determined using ELISA test kits (Wuhan Huamei Biotech co., LTD, Wuhan, china) in accordance with the manufacturer's instructions.

### Quantification of mRNA and cDNA synthesis by Real-time PCR analysis

The primers were designed with the use of Primer 5.0 according to the gene sequence of pigs to produce amplification products (Table 4). The expression of gene mRNA in the duodenum, jejunum and ileum was analyzed by Real-time quantitative PCR as described previously [47]. Relative gene expression was expressed as a ratio of the target gene to the control genes using the formula 2^−(ΔΔCt)^, where ΔΔ Ct = (Ct_Target_ – Ct _β-actin/GAPDH_)_treatment_ - (Ct_Target_ – Ct_β-actin/GAPDH_)_control_.

**Table 4:** Primers used for quantitative reverse transcription-PCR

Accession No.	Gene	Primers	Product length(bp)
XM_003124280.3	β-actin	F:CTGCGGCATCCACGAAACT	147
		R:AGGGCCGTGATCTCCTTCTG	
NM_001206359.1	GAPDH	F: AAGGAGTAAGAGCCCCTGGA	140
		R: TCTGGGATGGAAACTGGAA	
NM_001164021.1	SGLT-1	F:TCATCATCGTCCTGGTCGTCTC	144
		R:CTTCTGGGGCTTCTTGAATGTC	
NM_001097417.1	GLUT-2	F:GGTTCATGGTGGCCGAGTT	83
		R:ATTGCGGGTCCAGTTGC	
NM_214347.1	PEPT-1	F:CAGACTTCGACCACAACGGA	99
		R:TTATCCCGCCAGTACCCAGA	
NM_001031780.1	I-FABP2	F:TCGCAGACGGAACTGAACTC	131
		R:CTGGACCATTTCATCCCCGA	
NM_001163647.2	Occludin	F:TCAGGTGCACCCTCCAGATT	169
		R:TATGTCGTTGCTGGGTGCAT	
XM_005659811.1	ZO-1	F:CCAACCATGTCTTGAAGCAGC	215
		R:TGCAGGAGTGTGGTCTTCAC	
NM_001163060.1	E-cadherin	F:CAAACGGCCATTTCAGCTTCA	90
		R:GTCACCTTGGTGGACAGCTT	
NM_001244539.1	Claudin-1	F:AAGGACAAAACCGTGTGGGA	247
		R:CTCTCCCCACATTCGAGATGATT	
NM_001161638.1	Claudin-2	F:GCTGGCGAACGAGTTCTTAC	186
		R:AGATGGCGCTAGATGTCACC	
NM_001160075.1	Claudin-3	F:GCCAAGATCCTCTACTCCGC	195
		R:GAGAGCTGCCTAGCATCTGG	
XM_005661969.1	Claudin-4	F:TCAGCCCTGACTTTGCGTG	209
		R:ACCTGTCTGTCCACACCAC	
NM_001130861.1	Claudin-5	F:TCTGCTGGTTCGCCAACAT	259
		R:GACACCCTCAGACGTAGTTC	
XM_005662194.1	Claudin-6	F:TGTCTAATTCGGGTGGGGGT	88
		R:AAGCTGTCACGGATCACCG	
NM_001160076.1	Claudin-7	F:GACTTAAAATTCGCCCACCCG	217
		R:CGTGACGCAGTCCATCCATA	
NM_214055.1	IL-1β	F:GCTAACTACGGTGACAACAA	196
		R:TCTTCATCGGCTTCTCCACT	
NM_001252429.1	IL-6	F:CAAAGCCACCACCCCTAAC	66
		R:TCGTTCTGTGACTGCAGCTT	
NM_214013.1	IL-12	F: GCCAAGGTTACATGCCACAA	108
		R: TAGAACCTAATTGCAGGACACAGATG	

### Immunohistochemical analysis

The location of ZO-1, Occludin, and E-cadherin in jejunum and ileum of piglets were determined using Immunohistochemical analysis as described by He et al (2017) [24]. Sections were incubated with the anti ZO-1 (1:75; Proteintech Group, Inc., Los Angeles, CA, USA), Occludin (1:100; Abcam, Cambridge, LON, UK), E-cadherin (1:150; Proteintech group.,Inc.). The stained sections were scored independently by 2 investigators using a microscope at 400-fold magnification (Olympus, Tokyo, Japan).

### Western blot analysis

The expression of ZO-1, E-cadherin, Occludin, Claudin-1, and Claudin-2 proteins in the jejunum and ileum was determined by western blot analysis as described previously [16]. The following antibodies were used for protein quantification: ZO-1 (1:500; Proteintech Group, Inc.), Occludin (1:1000; Abcam), E-cadherin (1:2000; Proteintech Group, Inc.), Claudin-1 (1:1000; Cell Signaling Technology, Danvers, MA, USA), Claudin-2 (1:600; Abcam). All protein measurements were normalized to β-actin (1:4000; Proteintech Group, Inc.) and data are expressed relative to the values in control piglets.

### Statistical analysis

All statistical analyses were performed by factorial ANOVA using a mixed procedure (PROC MIXED) of SAS software version 9.2 (SAS Institute Inc., Cary, NC, USA). The statistical model included the effects of challenge (saline or LPS), diet (basal or AKG), and their interactions. All data were presented as Least Squares means plus pooled SEM. The Tukey multiple comparison test was used to evaluate the differences among the treatments. Probability values ≤ 0.05 were taken to indicate statistical significance.

## References

[1] Dokladny K, Zuhl MN, Moseley PL (2016). Intestinal epithelial barrier function and tight junction proteins with heat and exercise. J Appl Physiol.

[2] Fluck K, Fandrey J (2016). Oxygen sensing in intestinal mucosal inflammation. Pflug Arch Eur J Phy.

[3] Camilleri M, Madsen K, Spiller R, Van Meerveld BG, Verne GN (2012). Intestinal barrier function in health and gastrointestinal disease. Neurogastroent Motil.

[4] Hadi LA, Di Vito C, Riboni L (2016). Fostering Inflammatory Bowel Disease: Sphingolipid Strategies to Join Forces. Mediat Inflamm.

[5] Das MK (2008). Nutritional Management in Children with Inflammatory Bowel Disease. Indian J Pediatr.

[6] Wu LM, Ji JS, Yang Z, Xing CY, Pan TT, Xie HY, Zhang F, Zhuang L, Zhou L, Zheng SS (2015). Oncogenic role of microRNA-423-5p in hepatocellular carcinoma. Hepatob Pancreat Dis.

[7] Hou YQ, Wang L, Ding BY, Liu YL, Zhu HL, Liu JA, Li YT, Wu X, Yin YL, Wu GY (2010). Dietary alpha-ketoglutarate supplementation ameliorates intestinal injury in lipopolysaccharide-challenged piglets. Amino Acids.

[8] Dobrowolski P, Tomaszewska E, Bienko M, Radzki RP, Pierzynowski SG (2013). The effect of dietary administration of 2-oxoglutaric acid on the cartilage and bone of growing rats. Brit J Nutr.

[9] Wu N, Yang MY, Gaur U, Xu HL, Yao YF, Li DY (2016). Alpha-Ketoglutarate: Physiological Functions and Applications. Biomol Ther.

[10] Bayliak MM, Shmihel HV, Lylyk MP, Vytvytska OM, Storey JM, Storey KB, Lushchak VI (2015). Alpha-ketoglutarate attenuates toxic effects of sodium nitroprusside and hydrogen peroxide in Drosophila melanogaster. Environ Toxicol Phar.

[11] Hering NA, Schulzke JD (2009). Therapeutic Options to Modulate Barrier Defects in Inflammatory Bowel Disease. Digest Dis.

[12] Gassler N, Rohr C, Schneider A, Kartenbeck J, Bach A, Overmuller N, Otto HF, Autschbach F (2001). Inflammatory bowel disease is associated with changes of enterocytic junctions. Am J Physiol-Gastr L.

[13] Landy J, Ronde E, English N, Clark SK, Hart AL, Knight SC, Ciclitira PJ, Al-Hassi HO (2016). Tight junctions in inflammatory bowel diseases and inflammatory bowel disease associated colorectal cancer. World J Gastroentero.

[14] He LQ, Yang HS, Hou YQ, Li TJ, Fang J, Zhou XH, Yin YL, Wu L, Nyachoti M, Wu GY (2013). Effects of dietary l-lysine intake on the intestinal mucosa and expression of CAT genes in weaned piglets. Amino Acids.

[15] Zdzisinska B, Zurek A, Kandefer-Szerszen M (2017). Alpha-Ketoglutarate as a Molecule with Pleiotropic Activity: Well-Known and Novel Possibilities of Therapeutic Use. Arch Immunol Ther Ex.

[16] He LQ, Huang N, Li H, Tian JQ, Zhou XH, Li TJ, Yao K, Wu GY, Yin YL (2017). AMPK/alpha-Ketoglutarate Axis Regulates Intestinal Water and Ion Homeostasis in Young Pigs. J Agr Food Chem.

[17] Sliwa E (2006). Effect of simultaneous versus apart administration of dexamethasone and alpha-ketoglutarate on growth hormone, cortisol and insulin-like growth factor-I in piglets. B Vet I Pulawy.

[18] He LQ, Li H, Huang N, Tian JQ, Liu ZQ, Zhou XH, Yao K, Li TJ, Yin YL (2016). Effects of Alpha-Ketoglutarate on Glutamine Metabolism in Piglet Enterocytes in vivo and in vitro. J Agr Food Chem.

[19] Wang HQ, Zhao YR, Jin BT, Liang J (2016). Effects of Dietary Alpha-ketoglutarate Supplementation on Growth and Serum Biochemical Parameters of Grass Carp (Ctenopharyngodon idella) Fingerlings. Isr J Aquacult-Bamid.

[20] Opapeju FO, Rodriguez-Lecompte JC, Rademacher M, Krause DO, Nyachoti CM (2015). Low crude protein diets modulate intestinal responses in weaned pigs challenged with Escherichia coli K88. Can J Anim Sci.

[21] Ha TK, Lee GM (2014). Effect of glutamine substitution by TCA cycle intermediates on the production and sialylation of Fc-fusion protein in Chinese hamster ovary cell culture. J Biotechnol.

[22] Szefel J, Kruszewski WJ, Buczek T (2015). Enteral feeding and its impact on the gut immune system and intestinal mucosal barrier. Przeglad gastroenterologiczny.

[23] Xue HY, Sufit AJD, Wischmeyer PE (2011). Glutamine Therapy Improves Outcome of In Vitro and In Vivo Experimental Colitis Models. Jpen-Parenter Enter.

[24] He L, Li H, Huang N, Zhou X, Tian J, Li T, Wu J, Tian Y, Yin Y, Yao K (2017). Alpha-ketoglutarate suppresses the NF-kappaB-mediated inflammatory pathway and enhances the PXR-regulated detoxification pathway. Oncotarget.

[25] Garrett-Cox RG, Stefanutti G, Booth C, Klein NJ, Pierro A, Eaton S (2009). Glutamine decreases inflammation in infant rat endotoxemia. J Pediatr Surg.

[26] Li N, Liboni K, Fang MZ, Neu J (2004). Glutamine decreases inflammation in gastrostomy-fed infant rats. Pediatr Res.

[27] Chen S, Ren WK, Bin P, Yin J, Duan JL, Li YH, He LQ, Wu F, Liu G, Yao K, Yin YL (2015). Alpha-ketoglutarate lowers body weight and affects intestinal immunity through intestinal microbiota. Amino Acids.

[28] He L, Zhou X, Huang N, Li H, Li T, Yao K, Tian Y, Hu CA, Yin Y (2017). Functions of pregnane X receptor in self-detoxification. Amino Acids.

[29] Gabriel AS, Uneyama H (2013). Amino acid sensing in the gastrointestinal tract. Amino Acids.

[30] Wu L, Liao P, He LQ, Feng ZM, Ren WK, Yin J, Duan JL, Li TJ, Yin YL (2015). Dietary l-Arginine Supplementation Protects Weanling Pigs from Deoxynivalenol-Induced Toxicity. Toxins.

[31] Kellett GL, Brot-Laroche E, Mace OJ, Leturque A (2008). Sugar absorption in the intestine: The role of GLUT2. Annu Rev Nutr.

[32] Kalsi KK, Baker EH, Fraser O, Chung YL, Mace OJ, Tarelli E, Philips BJ, Baines DL (2009). Glucose homeostasis across human airway epithelial cell monolayers: role of diffusion, transport and metabolism. Pflug Arch Eur J Phy.

[33] He LQ, Niu H, Li H, Xu ZQ, Yao K, Li TJ, Yin YL (2016). Effects of dietary L-lysine supplementation on lysine transport by the piglet small intestine in vitro. J Anim Sci.

[34] Jaworski NW, Owusu-Asiedu A, Walsh MC, McCann JC, Loor JJ, Stein HH (2017). Effects of a 3 strain Bacillus-based direct-fed microbial and dietary fiber concentration on growth performance and expression of genes related to absorption and metabolism of volatile fatty acids in weanling pigs. J Anim Sci.

[35] Huang J, Zhang L, He CY, Qu Y, Li JF, Zhang JA, Du T, Chen XH, Yu YY, Liu BY, Zhu ZG (2015). Claudin-1 enhances tumor proliferation and metastasis by regulating cell anoikis in gastric cancer. Oncotarget.

[36] Li J, Li YX, Chen MH, Li J, Du J, Shen B, Xia XM (2015). Changes in the phosphorylation of claudins during the course of experimental colitis. Int J Clin Exp Patho.

[37] Chou HC, Chen CM (2017). Neonatal hyperoxia disrupts the intestinal barrier and impairs intestinal function in rats. Experimental and molecular pathology.

[38] Li X, Wang Q, Xu H, Tao LP, Lu J, Cai L, Wang CH (2014). Somatostatin regulates tight junction proteins expression in colitis mice. Int J Clin Exp Patho.

[39] Kucharzik T, Walsh SV, Chen J, Parkos CA, Nusrat A (2001). Neutrophil transmigration in inflammatory bowel disease is associated with differential expression of epithelial intercellular junction proteins. Am J Pathol.

[40] Wang B, Wu ZL, Ji Y, Sun KJ, Dai ZL, Wu GY (2016). L-Glutamine Enhances Tight Junction Integrity by Activating CaMK Kinase 2-AMP-Activated Protein Kinase Signaling in Intestinal Porcine Epithelial Cells. J Nutr.

[41] Wang H, Zhang C, Wu GY, Sun YL, Wang B, He BB, Dai ZL, Wu ZL (2015). Glutamine Enhances Tight Junction Protein Expression and Modulates Corticotropin-Releasing Factor Signaling in the Jejunum of Wean ling Piglets. J Nutr.

[42] Zheng YL, Zhang MQ, Zhao YM, Chen J, Li B, Cai WW (2014). JNK inhibitor SP600125 protects against lipopolysaccharide-induced acute lung injury via upregulation of claudin-4. Experimental and therapeutic medicine.

[43] Takehara M, Nishimura T, Mima S, Hoshino T, Mizushima T (2009). Effect of Claudin Expression on Paracellular Permeability, Migration and Invasion of Colonic Cancer Cells. Biol Pharm Bull.

[44] Bayliak MM, Lylyk MP, Shmihel HV, Sorochynska OM, Semchyshyn OI, Storey JM, Storey KB, Lushchak VI (2017). Dietary alpha-ketoglutarate promotes higher protein and lower triacylglyceride levels and induces oxidative stress in larvae and young adults but not in middle-aged Drosophila melanogaster. Comp Biochem Phys A.

[45] Akobeng AK, Elawad M, Gordon M (2016). Glutamine for induction of remission in Crohn's disease. Cochrane Db Syst Rev.

[46] He LQ, Xu ZQ, Yao K, Wu GA, Yin YL, Nyachoti CM, Kim SW (2015). The Physiological Basis and Nutritional Function of Alpha-ketoglutarate. Curr Protein Pept Sc.

[47] He L, Wu L, Xu Z, Li T, Yao K, Cui Z, Yin Y, Wu G (2016). Low-protein diets affect ileal amino acid digestibility and gene expression of digestive enzymes in growing and finishing pigs. Amino Acids.

